# Large diurnal bottom temperature oscillations around the Saint Pierre and Miquelon archipelago

**DOI:** 10.1038/s41598-018-31857-w

**Published:** 2018-09-17

**Authors:** Pascal Lazure, Bernard Le Cann, Marion Bezaud

**Affiliations:** Ifremer, Univ. Brest, CNRS, IRD, Laboratoire d’Océanographie Physique et Spatiale (LOPS), IUEM, Brest 29280, France

## Abstract

Here, we report large, near-daily oscillations of near-bottom temperatures, with ranges of up to 11.5 °C at depths of 30–60 m in September 2011 around the Saint Pierre and Miquelon archipelago (south-eastern Canada). These oscillations were detected on velocity and temperature profiles from moorings in Miquelon Bay and on an array of near-bottom temperature sensors around the archipelago. The oscillations coincided with the seasonal stratification period. In addition to their remarkable range, they exhibited a near-diurnal period centred on the O1 tidal component (~26 h), contrasting with the dominant semi-diurnal sea-level periodicity in the area. They appear to be the manifestation of an internal wave, triggered by the diurnal surface tide and trapped by the bathymetric configuration of the area. We argue that the archipelago is nearly resonant for island-trapped waves at near-diurnal frequencies. Our data demonstrate that these coastal-trapped waves propagate clockwise around the archipelago in roughly two days, and thus approximate an azimuthal, mode 2 pattern. Simplified calculations show that cross-shore motions are bottom-amplified. In addition, bottom friction acts to rotate the axes of the diurnal tidal current ellipses with depth, and this combination of effects explains the large range of observed bottom temperature oscillations.

## Introduction

Large seabed temperature oscillations have been observed in shallow waters at many locations around the world. They generally occur in stratified environments and have a broad, high-frequency spectrum typically ranging from several hours to a few days. Strong seabed temperature fluctuations with ranges of 6–8 °C have been reported around reefs or submarine cliffs with variable frequencies: mainly semi-diurnal frequencies near the French Polynesian islands^1^, mixed diurnal and semi-diurnal frequencies in the South China Sea^2^ and in the Adriatic Sea^3,4^ or at subtidal frequencies in the Andaman Sea^5^. In the nearshore region of broad continental shelves, seabed temperature fluctuations are generally weaker. To date, the largest documented oscillations include a recent report of near-tidal-front fluctuations of up to 7 °C with a semi-diurnal frequency at depths of around 60 m over Georges Bank (North-west Atlantic Ocean)^6^ and diurnal and semi-diurnal frequency fluctuations of 6 °C at 15 m depth along the Californian coast^7^.

Several explanations have been proposed for these observations, depending on the frequency of the oscillations, either above or below the local Coriolis frequency. At sub-inertial frequencies, coastal-trapped waves (CTWs)^8^ are often cited. When stratification is negligible, oscillations can be attributed to barotropic continental shelf waves (CSWs), for which topography provides the restoring mechanism. On the other hand, under sufficiently strong stratification, topography may be neglected and waves become very similar to Kelvin waves (KWs), where the sloping bottom has a purely geometric effect. CTWs may be bottom-intensified^9^, which enhances their near-bottom impact.

Regarding the forcing of these waves, tides and wind are typically cited as factors. (Nearly) periodic oscillations are frequently attributed to tides, although diurnal sea breezes can also induce periodic upwelling^10–13^.

At mid-latitudes, diurnal tides are sub-inertial, and strong currents have been frequently reported, e.g. in the North-east Atlantic Ocean^14–16^, on the west coast of Vancouver Island^17,18^, near the Kuril Islands (North Pacific Ocean)^19^ and other locations often characterised by curved shelf breaks on a scale of 10–100 km. These currents are typically attributed to CTWs^20^.

A special case is when the area of interest shows closed topographic contours, such as a seamount or an island. These cases of “island-trapped waves” (ITWs) have been studied using theoretical approaches^21–24^. They open the possibility of resonant forcing and amplification of CTWs around a seamount or island^3,4,25^.

**Figure 1:**
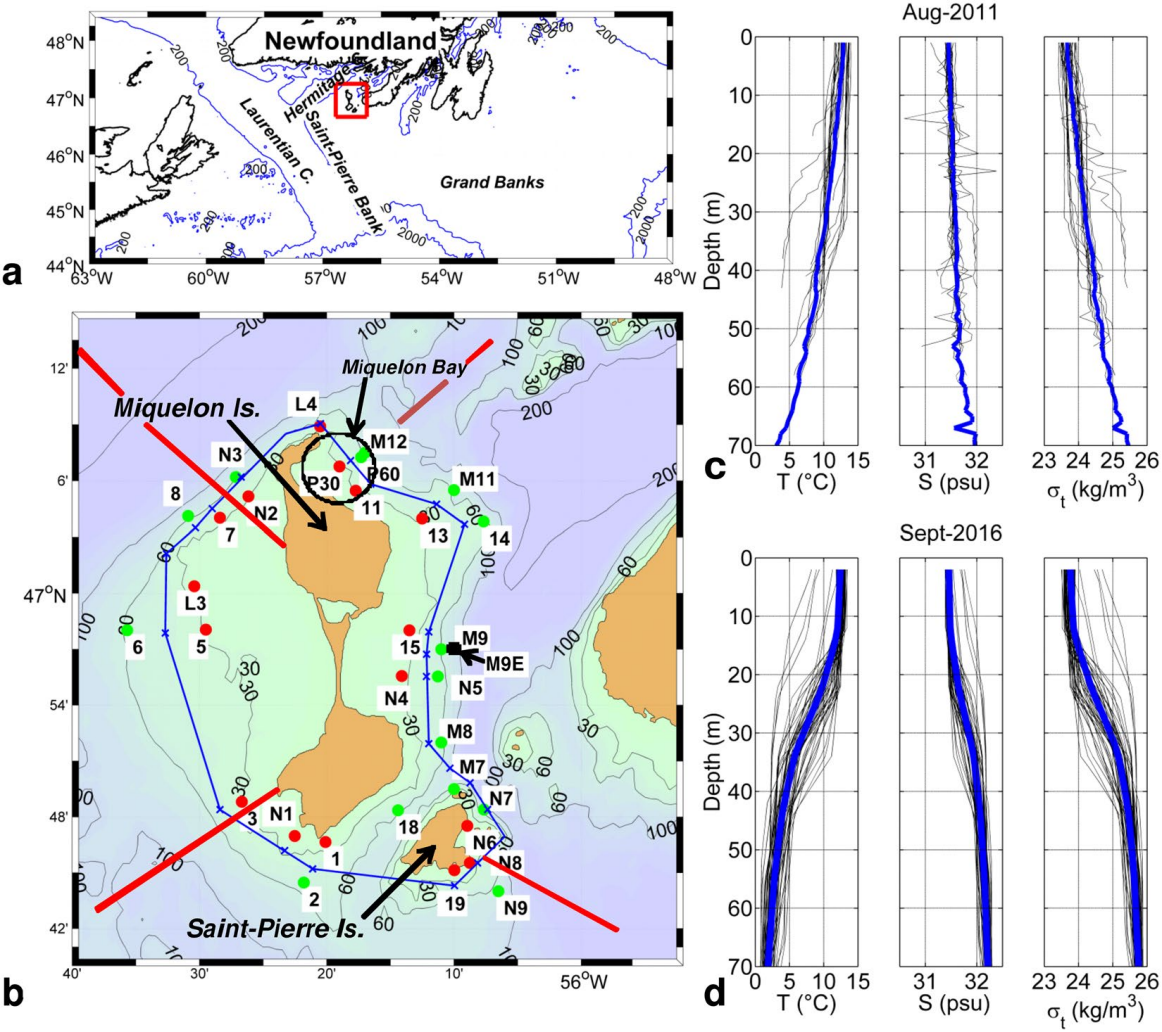
(**a**) Map and bathymetry south-west of Newfoundland. (**b**) Map and bathymetry of the Saint Pierre and Miquelon (SPM) archipelago. Red and green dots respectively indicate the moorings at 30 and 60 m depth deployed in 2011, 2015, 2016 and 2017 (see Methods for details). The blue line roughly follows the 45 m isobath and is used as the along-shore curvilinear axis. The location of each station along this axis is indicated with a blue ‘x’. The four red lines indicate the transects used in the schematic modelling (see discussion). (**c**) Temperature, salinity and density profiles in the Miquelon Bay measured within a polygon defined by Stations L4, M11, 11, P30 in August 2011. Thick blue lines show mean profiles. (**d**) Temperature (T), salinity (S) (calculated according to a T/S relationship, see Methods) and density profiles shown every 3 h at Station M9E measured from the surface to the bottom during one week in September 2016 using a mooring with ten temperature and pressure sensors. Thick blue lines show mean profiles. The maps were generated using M_MAP_V1.4 package for Matlab (http://www.eos.ubc.ca/~rich/map.html).

Here, we report exceptionally large near-bottom diurnal temperature oscillations of up to 11.5 °C around the Saint Pierre and Miquelon (SPM) archipelago, located south-west of Newfoundland (Fig. 1), at the north-west edge of its large continental shelf. These direct observations are based on the recordings from ADCP current meters and thermistors deployed in Miquelon Bay in 2011 (Stations P30 and P60). Further experiments were conducted during the stratified period of 2015, 2016 and 2017 with a dense array of near-bottom temperature sensors distributed around the archipelago, along the 30–60 m isobaths and two ADCPs in summer 2015. This bottom array provided a detailed overview of the spatial and temporal characteristics of the oscillations. In addition, we also deployed a mooring fitted with 10 thermistors from the surface to the bottom during one week in September 2016 (the data set and characteristics of all instruments are presented in Methods, Table 1).

**Table 1:** Data used in this study.

Mooring Station	water depth (m)	Instrument	Instrument depth (mab)*	Sampling period
P30	30	ADCP RDI 600 kHz	0.5, 1 m bins	22 Jul to 1 Nov 2011
P30	30	CTD (NKE)	5, 25	1 May to 1 Nov 2011
P30	30	CTD (NKE)	1,16	22 Jul to 1 Nov 2011
P60	60	CTD (NKE)	5, 55	1 May to 1 Nov 2011
1, 3, 5, 7	30	Mastodon (near-bottom temperature measurement, see Methods)	0.5	4 Jul to 15 Sep 2015
11, 13, 15, 19	30	Mastodon	0.5	4 Jul to 14 Nov 2015
2, 6, 8	60	Mastodon	0.5	4 Jul to 15 Sep 2015
14, 18	60	Mastodon	0.5	4 Jul to 14 Nov 2015
L3	30	ADCP RDI 600 kHz	0.5, 1 m bins	2 Jul to 15 Sept 2015
L4	28	ADCP RDI 600 kHz	0.5, 1 m bins	17 Sep to 10 Oct 2015
M7, M8, M9, M11, M12	60	Mastodon	0.5	13 Jul to 30 Oct 2016
M9E	75	East Mastodon	0.5, 5, 11, 21, 31, 41, 51, 62, 72, 75	9 Sep to 16 Sep 2016
N1, N2, N4, N8	30	Mastodon	0.5	13 Aug to 14 Sep 2017
N6	30	Mastodon	0.5	26 Aug to 13 Sep 2017
N3, N9	60	Mastodon	0.5	13 Aug to 14 Sep 2017
N5, N7	60	Mastodon	0.5	26 Aug to 13 Sep 2017

*mab, meters above the bottom.

We first present our observations and results, and then our analysis in support of an ITW propagation around the archipelago.

## Results

### Observations on the northeast shelf of Saint-Pierre and Miquelon

During the summers and early fall of 2011, 2015 and 2016, waters of the northeast shelf of SPM were seasonally stratified in temperature and salinity. Density differences from surface to bottom, mostly due to temperature, varied from ~1.9 kg/m^3^ in August 2011 (Fig. 1c) and September 2016 (Fig. 1d) to ~1.4 kg/m^3^ in October 2011 (not shown).

**Figure 2:**
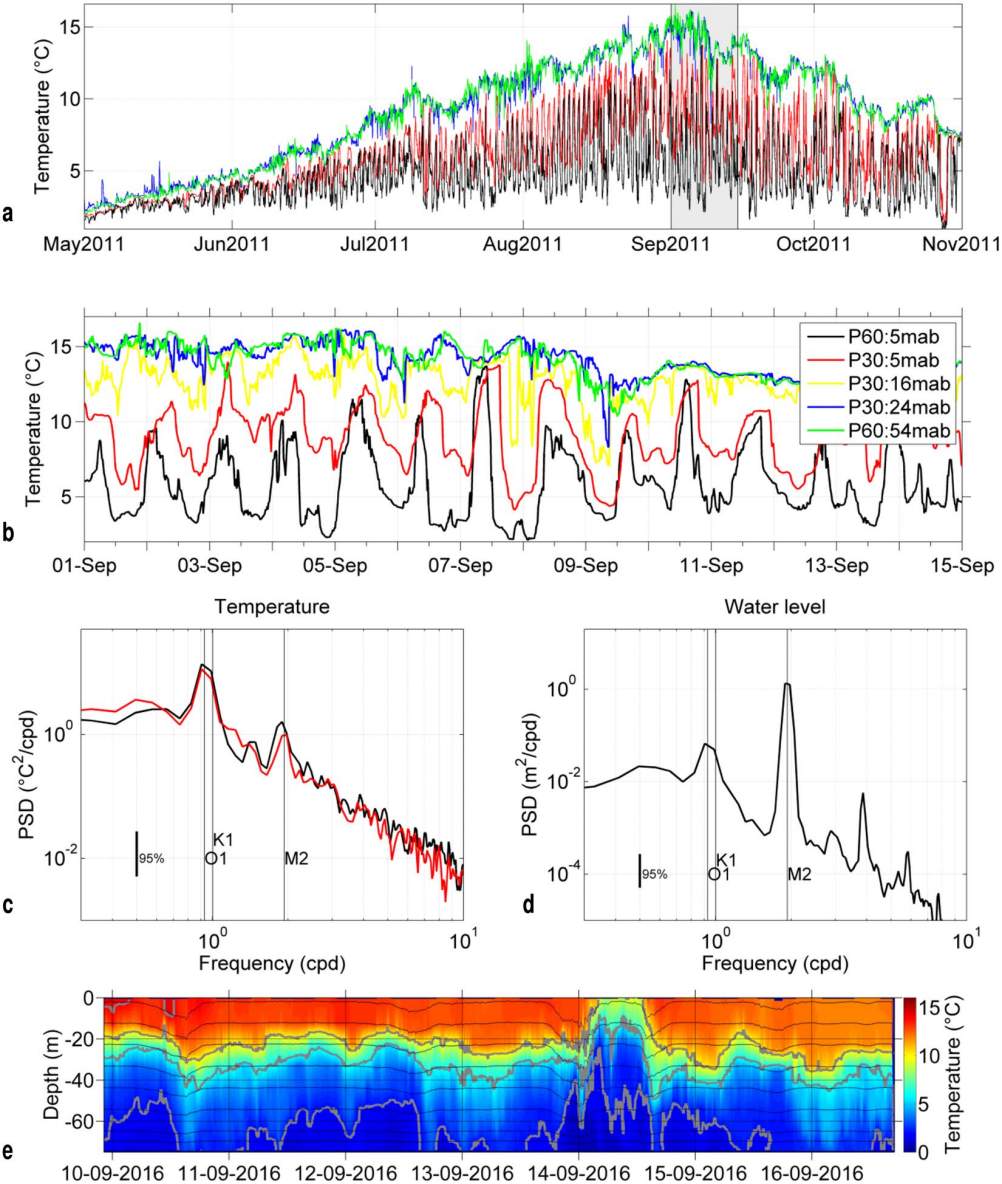
(**a**) 2011 time series of temperature at 6 m below the surface (blue and green) and 5 m above the bottom (red and black) at Stations P30 and P60, respectively. (**b**) Same as (a) with a close-up on the first two weeks of September (shaded in (a)) and mid-depth temperature recording at P30 (16 m above the bottom (mab)). (**c**) Spectral analysis of bottom temperature from 01 Aug 2011 to 15 Oct 2011 at Stations P30 (red) and P60 (black). (**d**) Spectral analysis of bottom pressure recorded at Station P30 (same period). (**e**) Time evolution of temperature at station M9E in September 2016. Temperatures have been vertically interpolated every 2 m and decimated to a time step of 5′. Thin black lines show the depth of thermistors. Grey lines show the isotherms 2,6,10 and 14 °C.

The May-to-November 2011 time series of temperature at 6 m below the surface and at 5 m above the bottom (mab) at Stations P30 and P60 are shown in Fig. 2a. Near the surface, the temperature varied from 2 °C in mid-spring to a maximum of 15 °C in early September and then decreased to 8 °C in November. However, near the bottom, the temperature variations were radically different. Bottom temperatures showed high-frequency variations whose amplitude increased with sea-surface temperature. Over the whole study period, the minimum temperature remained close to 2–3 °C and showed little seasonal variation. During the first two weeks of September (Fig. 2b), oscillations showed large amplitude, reaching 11.5 °C at Station P60 and 9.5 °C at P30 (7 September). Interestingly, in addition to this large amplitude, the oscillations featured a daily period, whereas the local sea-level tide is mostly semi-diurnal with a mean range of 1.3 m (not shown). To identify the dominant frequencies, a spectral analysis was performed on bottom temperature (Fig. 2c) and sea level (Fig. 2d). The dominant frequency for temperature variability was 0.93 cycles per day (cpd), which corresponds to the O1 tidal component with a period of 25.82 h (see also harmonic analysis results in Supplementary Table S1). The second identified periodicity was 1.00 cpd which corresponds to the K1 component, whereas the M2 component (1.93 cpd) was an order of magnitude weaker. Conversely, sea-surface elevation (Fig. 2d) showed a dominant semi-diurnal period.

At Station P30 (Fig. 2b), the temperature variability at different depths increased from the surface to the bottom. The daily drop of the near-bottom temperature showed high day-to-day variability. The cold phase near the bottom generally affected almost the entire water column, but was only slightly noticeable near the surface. The rise and fall of bottom temperature reached up to 6 °C/h and was not symmetric: the cooling phase was generally longer than the warming phase. Rapid warming generally led to a nearly homogeneous water column with bottom temperatures similar to that of the surface for about 1 to 6 h. Conversely, around the daily minimum, we rarely observed stable cold temperatures for more than 1 h.

To further describe the time evolution of vertical stratification on hourly to weekly time scales, we examined the vertical thermal structure data in station M9E (Fig. 1b) collected in September 2016. On such a short record, it is difficult to ascertain periodicities, but there are indications of isotherms vertical oscillations of roughly semi-diurnal and diurnal periods (Fig. 2e). There are even hints of a nearly two-day period. A notable episode of strong isotherms uplifting took place on 14 September. This event does not seem to be linked to local wind forcing, as wind speed at the nearby Saint Pierre airport (not shown) was maximum at ~12 m/s on 12 September to decrease at 4–8 m/s on 14 September. Apart from this episode, isotherms oscillation amplitudes ranged from ~20 m near the bottom, to ~10 m at thermocline levels, and nearly vanished near the surface. Overall, at this deeper station, diurnal scale bottom temperature oscillations were weaker than the oscillations observed at stations P30 and P60 (Fig. 2b), and less well defined.

**Figure 3:**
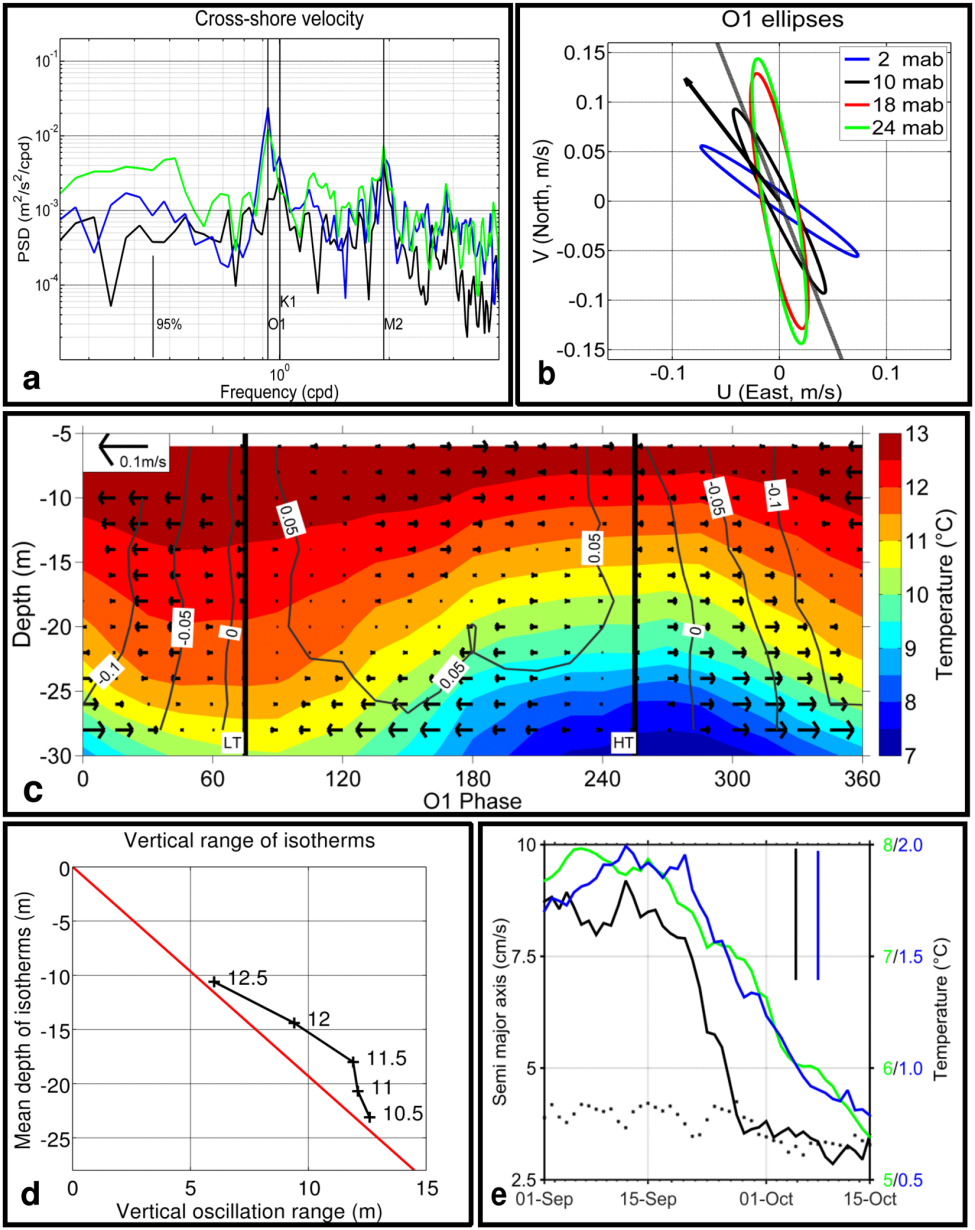
Current and temperature at Station P30 (see Fig. 1 for location) for the period running from 01 Aug 2011 to 30 Sept 2011. (**a**) Spectral analysis of cross-shore currents: near-bottom current, 2 mab (blue line), depth-averaged current from 2 to 24 mab (black line), near-surface current, 24 mab (green line). (**b**) Tidal ellipses for the O1 tidal component at different depths as indicated in the figure key. The black arrow indicates the mean depth-averaged current; the grey line indicates the along-shore axis after rotation (see Methods). (**c**) O1 phase-averaged temperature (colour scale on the right) and rotated cross-shore currents (vectors pointing to the right denote offshore flow). Labelled isolines denote rotated along-shore currents (positive is northward). HT, diurnal high tide; LT, diurnal low tide. (**d**) Observed mean ranges of vertical oscillations for selected isotherms derived from Fig. 3c (black crosses). The red line gives the linear range predicted from the kinematic bottom and surface boundary conditions (see Discussion for details). (**e**) Time evolution of semi-major axis (left Y axis) calculated for the O1 (black line) and M2 (dotted black line) tidal components (2 mab). Time evolution the O1 tidal component of near-bottom (5 mab) temperature at P30 (blue line, right Y axis) and difference between near-surface and near-bottom temperatures at P60 (green line, right Y axis). Vertical black and green lines show the average error calculated by t_tide for the O1 component of current and temperature, respectively. Harmonic analysis was performed over sliding periods of 30 days and results plotted at mid-periods.

The simultaneous ADCP observations at Station P30 in Miquelon Bay were used to help interpret these bottom temperature oscillations. The mean current in August-September (Fig. 3b) pointed to the north-west and was relatively strong (~15 cm/s). A rotation of 20° in the anticlockwise direction was applied (see Methods for details) to screen for the best correlation between cross-shore transport and temperature variability. This direction roughly follows the local isobaths.

Along these new axes, the current spectra showed two pronounced peaks with dominant diurnal and semi-diurnal frequencies (Fig. 3a). In the cross-shore direction (70° clockwise from North), the diurnal frequency dominated, with O1 currents larger than K1 currents. M2 currents were even weaker. This O1 dominance was confirmed by the tidal harmonic analysis of currents at different depths (Supplementary Table S1). At semi-diurnal frequencies, cross-shore currents were surface-intensified, but at diurnal frequencies, cross-shore currents were slightly bottom-intensified (Fig. 3a).

In the along-shore direction, the pattern was similar (not shown) with a notable exception: the near-bottom currents were not intensified and the near-surface currents dominated at diurnal frequencies.

Considering the dominant O1 tidal component, currents (band-pass filtered from 1 to 30 h to focus on high-frequency motions) and temperature were time-averaged over two months (August and September) according to the O1 phase, with phases 255° and 75° corresponding to the diurnal high and low tide, respectively (Fig. 3c). The bottom flow currents lasted about 12 h, bringing cool water from the south-east in the bottom layer. After a short slack period around high tide, the near-bottom ebb currents reached ~5 cm/s and flushed the cold bottom water out. The bottom temperature then increased by ~5 °C. Meanwhile, surface cross-shore currents were directed onshore, thickening the warm surface layer and establishing vertical shear. In the along-shore direction, the currents strengthened during the ebb tide and reached ~15 cm/s in the southward direction near the surface.

The major axes of the O1 tidal ellipses (Fig. 3b) decreased and rotated anticlockwise with increasing depth. The major axis of the mid-depth O1 ellipse was roughly aligned with the along-shore isobaths. O1 ellipses were polarised clockwise and Greenwich phases for O1 currents were almost constant with depth (139° + −3°, see Supplementary Table S1). Given that semi-major axes rotated with depth (parameter ‘Inc’ in Supplementary Table S1), and pointed to one side of the along-shore axis near the bottom, and to the other side of the along-shore axis near the surface, the cross-shore surface and bottom currents were roughly out of phase (Fig. 3c). The small phase shift of the cross-shore current structure with depth can be attributed to the ellipses that rotated with depth, with a clockwise polarisation. Similar results were obtained for the O1 ellipses in 2015 at the L3 and L4 ADCP stations, although the isobath orientations were different (not shown, but see Supplementary Table S1).

Maximum temperature fluctuations occurred near bottom, but were also clearly seen in the water column. The range of the vertical oscillations of selected isotherms, whose entire O1 tidal cycle can be observed in Fig. 3c, was estimated (Fig. 3d). Maximum range (12.5 m) was obtained for the isotherm closest to the bottom (isotherm 10.5 °C at 23 m mean depth), compared with local depth of 30 m. Ranges for the shallower isotherms then decreased away from the bottom, down to a range of 6 m for isotherm 12.5 °C at 11 m mean depth.

The steep and abrupt decrease in the amplitude of the diurnal currents during the second half of September 2011 was quite pronounced (Fig. 3e). From a value of ~8 cm/s during the first half of September, the semi-major axis of the O1 component of the near-bottom current fell to ~3 cm/s during the first half of October, i.e. in ~10 days. The same behaviour was seen in the O1 component of the surface current (not shown), but not in the semi-diurnal component (M2), which remained stable during the period (Fig. 3e). A much weaker (~3 cm/s to ~2 cm/s) decrease of semi-major axis of K1 component was also observed (not shown). This decrease in the O1 current also affected the O1 component of bottom temperature, whose amplitude fell from ~1.9 °C during the first half of September to ~0.8 °C by mid-October. These changes are related to the seasonal decrease in vertical temperature stratification. The difference between the surface and bottom temperatures at P60 decreased from ~8 °C in early September to ~5.5 °C in mid-October (Fig. 3e).

### Observations of bottom temperature over the whole archipelago

To assess the diurnal bottom variability across the whole archipelago compared with that observed in Miquelon Bay in 2011 at P30 and P60, an array of 29 bottom temperature sensors (0.5 mab) was deployed on the shelf around the archipelago in 2015, 2016, 2017 and temperature sensors of ADCP moorings L3 and L4 were used in summer and early fall 2015 (see Fig. 1a and Table 1 for the characteristics of the whole data set). Strong diurnal oscillations were observed at all 13 stations in 2015, and are described below.

**Figure 4:**
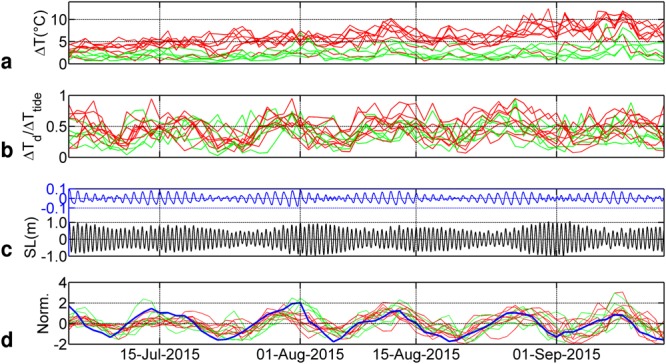
(**a**) Daily range of bottom temperature records (15 h before and after noon each day) at the 13 stations (labelled from 1 to 19, see Fig. 1 for their locations) in 2015 (30 m and 60 m depth in red and green, respectively). (**b**) Ratio of the diurnal band-passed (20–30 h) range to the high frequency range (1–30 h). (**c**) Sea level (SL) recorded near Miquelon (black line). Diurnal band-passed (20–30 h) sea level is shown in blue (y scale is stretched). (**d**) Normalised daily diurnal range for bottom temperature (green and red) and daily range of band-passed diurnal sea level oscillations (envelope of diurnal signal in (**c**) (thick blue line)).

The daily temperature range (difference between maximum and minimum temperatures during a 30 h interval centred around noon) of the 13 moorings is shown in Fig. 4a. At depths of 60 m, the range was around 3 °C and increased to an average value of 6 °C at depths of 30 m. In Miquelon Bay, although the temperature range at 60 m was on average smaller than at 30 m in 2011 (Fig. 2a,b), the highest daily range (11.5 °C) was observed at 60 m on 7 September 2011 (but not in 2015, as shown in Fig. 4a). This anomaly is somehow an indication of higher (than diurnal) frequency phenomena, and is probably also related to interannual differences, notably of mean vertical stratification and thermocline depth. We lack proper estimations of these latter parameters in 2015. A seasonal increase in variability was also observed from the beginning of July to mid-September, linked to summer surface warming. The daily range was also computed for the same time series, filtered around the high-frequency band (1–30 h) and around the diurnal frequency band (20–30 h). Their ratio (Fig. 4b) indicates that diurnal oscillations accounted for around half of the total variance of the signal regardless of the depth of the moorings. To highlight the potential causes of temporal change in the amplitude of diurnal oscillations, the recorded tide level is shown (Fig. 4c) and the diurnal component was extracted with a band-passed filter (20–30 h). The bottom temperature observations were also harmonically analysed (Supplementary Table S2). At all stations, the observed predominant periodicity was that of the O1 tide.

The classical spring-neap tidal cycle of the total sea-level record has a period of 14.8 days, due to the beat modulation of the two main semi-diurnal components (M2 and S2). However, there is also a spring-neap tidal cycle at diurnal frequencies: a beat modulation of the main diurnal waves (O1 and K1) with a period of 13.7 days (Fig. 4c). This latter period appeared in the bottom temperature records. The normalised diurnal bottom temperature range and the daily range of the diurnal band-passed sea level fluctuated synchronously (Fig. 4d), providing further evidence that the diurnal tide is the main driver of the diurnal seabed temperature fluctuations and can modulate its range depending on the range of the diurnal tide sea-level oscillation.

## Discussion

At the local scale of the SPM archipelago, we compiled comprehensive evidence for strong near-diurnal current and bottom temperature oscillations. Sea breezes can produce strong diurnal oscillations, as shown for example along the Californian coast^10^. However, in the SPM region, the diurnal signal in the wind spectrum was weak (not shown), strongly suggesting that wind is not likely to be the root cause of the phenomenon and in any case cannot explain the dominant ~26 h period.

The current and temperature oscillations displayed a peak at the O1 tidal frequency, suggesting tidal forcing. At a regional scale encompassing the SPM region, high-frequency tide-related hydrodynamics has already been studied using numerical models^26–28^, showing that the tide is mainly semi-diurnal with a dominant M2 wave. At Saint Pierre Harbour, the M2 amplitude is 60 cm whereas O1 and K1 diurnal waves are respectively 6 cm and 7 cm^27^. However, diurnal tidal currents (K1 and O1) are locally amplified in the vicinity of the Grand Banks slope and near SPM^28–30^. This feature is attributed to a mode 1 CSW at a diurnal frequency and possibly to the propagation of double KW along the slope^30^.

At the SPM latitude (~47°N), diurnal oscillations are sub-inertial and cannot propagate freely. The combined influence of topography and stratification may give rise to CTWs^8^ propagating with the coast on their right in the northern hemisphere. The influence of stratification on CTWs can be estimated with the Burger number (S = (NH/fL)^2^, where N is the Brunt-Väisälä frequency, H and L the vertical and horizontal scales and f the Coriolis frequency). Small values of S imply weak stratification effects and CSW-type waves, whereas large values of S indicate that the trapped waves are more similar to internal KWs, with the sloping bottom having only a geometric effect^8^. Around islands or seamounts, these waves may give rise to ITWs^21,22^. Other observations have attributed diurnal oscillations to ITW-type waves^25^; a recent example is the diurnal oscillations reported in the Adriatic Sea^4^.

In Miquelon Bay and along the eastern side of Miquelon Island (Fig. 1c,d), the density difference between the surface and bottom along the 60 m isobaths was estimated at ~2 kg/m^3^ giving a depth- averaged N ~0.02 s^−1^. The density profile in 2016 (Fig. 1d) showed a nearly homogenous surface layer above a smooth thermocline between 20 and 40 m in which N reached ~0.06 s^−1^. The bottom slope (H/L) between the 20 and 100 m isobaths was ~10^−2^ in the cross-shore direction. We thus obtained an average value of S of ~4, indicating that, in our case, stratification plays a significant role in wave characteristics.

To test the hypothesis of the propagation of anomalies as CTWs/ITWs, and particularly the phase evolutions, we examined the harmonic analysis results (using the t_tide Matlab package^31^) including the four dominant O1, K1, M2, S2 waves of the temperature time series (Supplementary Table S2). The harmonic analysis gave its best results at Stations 6, 7, P60 and 19 where more than 50% of the total variance in bottom temperature was explained by the sum of the tidal components. The diurnal O1, K1 and the semi-diurnal M2 components dominated, the S2 component being weaker.

Station-averaged amplitudes for the 31 analysed stations were 1 °C, 0.55 °C and 0.35 °C for the O1, K1 and M2 components, respectively. Maximum amplitude for the O1 component of 3.0 °C (Supplementary Table S2) was observed in the northwest at Station 7. Amplitude for the K1 component showed the same spatial pattern. M2 amplitudes were noticeably larger along the eastern side of Miquelon Island.

**Figure 5:**
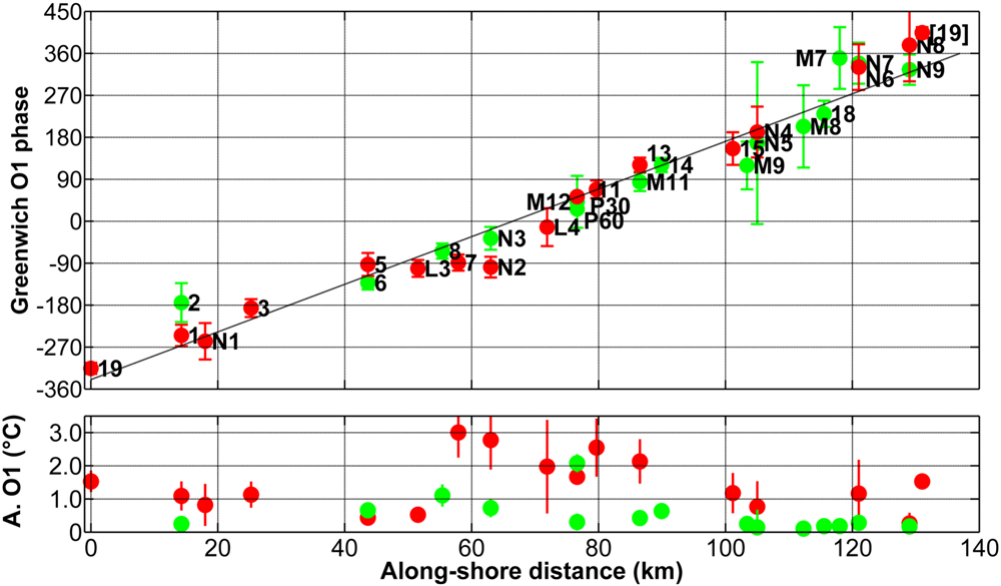
(upper panel) Greenwich O1 phases of the harmonic analysis of bottom temperature along the curvilinear axis around the SPM archipelago (blue line in Fig. 1). Red and green circles indicate the moorings at 30 and 60 m depths deployed in 2011, 2015, 2016 and 2017. The origin of the axis was arbitrarily set at Station 19 (SE of Saint Pierre Island) and distances were computed in the clockwise direction. Phases of Stations 19 to L4 were shifted by −360°. The labels indicate the name of the station (see Fig. 1 for location). Vertical lines indicate the phase error, with a 95% confidence interval, as estimated by t_tide. A linear fit was computed (black line), R^2^ = 0.95. [19] is the phase at Station 19 shifted by + 360°. Station 19 was only used once (at x = 0) for the linear fit. Lower panel: Amplitude of the O1component in the harmonic analysis of bottom temperature. Vertical lines indicate the amplitude error.

The variations of the O1 phase between the stations displayed an interesting pattern. As seen in Fig. 5, the phases increased clockwise. Arbitrarily taking Station 19 as the origin, there was a 2 * 2π phase increase along the curvilinear axis (see Fig. 1). Although the analysis is based on different years of measurements (see Table 1 in Methods) in summer, the obtained pattern appears very consistent. From the linear fit (Fig. 5), the phase speed was estimated at 0.81 m/s. Some discrepancies appear, notably for stations for which the O1 amplitude and explained variance (Supplementary Table S2) were relatively small, e.g. Stations 2 and M7. Amplitude estimation both in space and time is less consistent than phase determination, as it depends on many factors, like the seasonal thermocline mean gradient and depth on different periods of different years, and on relative position of temperature sensor and thermocline.

**Figure 6:**
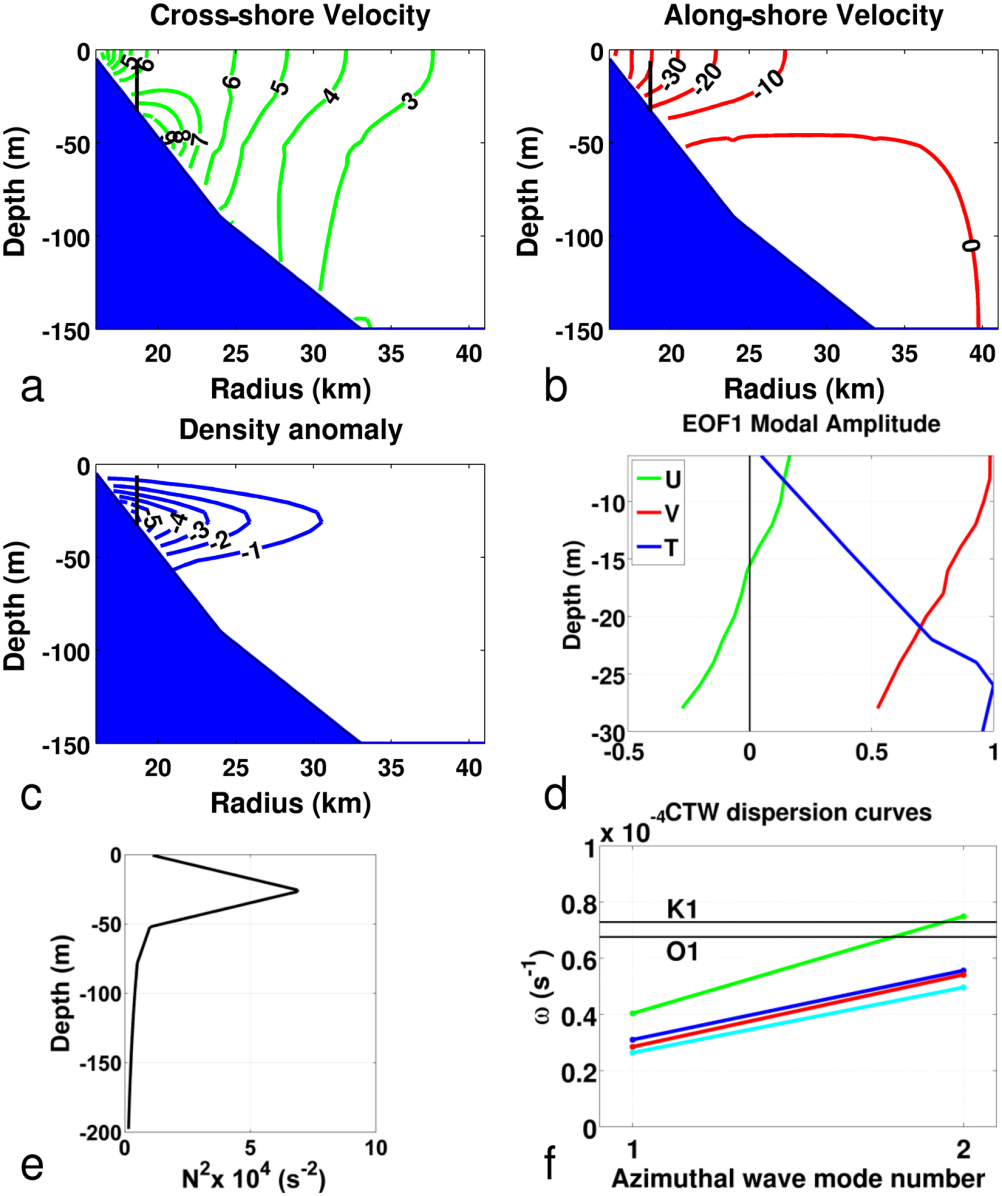
(**a**–**c**) Across-shelf modal structure (azimuthal mode 2) for a schematic north-east cross-shore transect representing Miquelon Bay computed using Brink’s programs^32^. (**a**) Cross-shore velocity, (**b**) Along-shore velocity, (**c**) Density anomaly. The short black vertical line at ~30 m depth in (**a**–**c**) approximates the location and vertical span of the P30 mooring. (**d**) First EOF mode structure computed from observations in P30 (green: cross-shore velocity, red: along-shore velocity, blue: temperature). (**e**) N^2^ profile used for computations resulting in (**a**–**c**) and (**f**). (**f**) Dispersion diagram around Saint Pierre and Miquelon archipelago using four different bathymetric transects (green: north-west, blue: north-east (Miquelon Bay), cyan: south-east, red: south-west). The two black horizontal lines indicate the K1 and O1 frequencies.

To gain further insight, we used the numerical model developed by Brink^32^ to compute the ITW properties around the SPM archipelago. The model assumes an exact azimuthal symmetry. We used the density profiles estimated at Station M9E in September 2016 (Fig. 6e). The dispersion diagram for the first two azimuthal modes of ITWs was computed for four bathymetry transects around SPM (Fig. 6f). The azimuthal wave number 2 (i.e. two wavelengths around the archipelago) ITW roughly corresponds to diurnal frequencies. Modal structure of cross- and along-shore velocities and density anomalies for this azimuthal wave number 2 are plotted in Fig. 6a–c, for the north-east (Miquelon Bay) transect. Compared with the first dynamical mode computed from the observed vertical structure of velocity and temperature (a proxy for density) profiles, we noted similarities in computed and observed anomalies in cross-shore and along-shore velocities and density/temperature structures. With an archipelago perimeter of around 130 km, we obtained phase speeds in the range 0.5–0.8 m/s. Given the approximations involved in the computations, calculated phase speeds were relatively similar to the observed speeds. We thus interpret our observations as the manifestation of a diurnal azimuthal mode 2 ITW propagating around the archipelago. Azimuthal resonance is far from perfect though, because SPM is not a cylindrical island, because cross-shore topography is not azimuthally uniform.

In the framework of this ITW interpretation, we may also understand the abrupt decrease in amplitude of O1 currents in the second half of September 2011 (Fig. 3e): as vertical density stratification decreases, so does the phase speed of the CTWs^8^. We may thus rapidly move away from the azimuthal resonance conditions for O1. As K1 component was already weaker and away from resonance, its decrease is less influenced by vertical density changes.

In these shallow waters, bottom friction and bottom Ekman layers also play a role, as demonstrated by the rotation of the axes of current ellipses with depth, by ~40° in ~30 m (Fig. 3b) as expected with typical vertical eddy viscosity and linear bottom friction coefficients^33,34^ (respectively Kv~10–30 cm^2^/s and r~0.05 cm/s). This rotation of the ellipses with depth can enhance the cross-shore flow near the bottom, in conjunction with a near-surface opposite cross-shore flow.

We thus interpret the vertical oscillations of the isotherms (Fig. 3d) as the combined result of the kinematic boundary condition on near-bottom currents, rotated by friction, and of baroclinic effects induced by the ITW. At the bottom (z = −H), the boundary condition implies that the vertical velocity ${w}_{-H}={u}_{-H}\partial H/\partial x$, where *u*_*−H*_ is the cross-shore bottom velocity and $\,\partial H/\partial x$ is the cross-shore bottom slope. At the surface (z = 0), vertical velocity is w(0)~0. We checked from our observations that, at first order, the temporal variations of bottom temperature ($\partial T/\partial t)$ are induced by vertical advection (w_−H_) of the mean near-bottom vertical gradient of temperature ($\overline{\partial T/\partial z}\,$): $\partial T/\partial t \sim -{w}_{-H}\overline{\partial T/\partial z}$. In the water column, the barotropic part of the vertical velocity w(z) (respectively vertical displacements d(z)) of isotherms induced by the kinematic bottom boundary condition goes linearly from w_−H_ (respectively maximum displacement dmax) at the bottom to ~0 at the surface. Thus, w(z) = −z/H ${{\rm{w}}}_{-{\rm{H}}}$ (respectively d(z)=−z/H dmax) (see the plot of d(z) in Fig. 3d, with *u*_*−H*_ = 0.05 m/s and $\partial H/\partial x$=0.01, giving a dmax of ~15 m). The vertical displacements of the two isotherms 10.5 °C (closer to bottom) and 12.5 °C (closer to surface) are well in line with this estimation. However, the displacements of the three intermediate isotherms displayed a slight excess in vertical displacement from the linear d(z), which we interpret as enhanced near-bottom baroclinic effects.

A possible generation mechanism may be linked to the propagation in the area of a mode 1 CSW originating on the western continental slopes of the Labrador Sea^28,30^. Along-shore topography abruptly changes north-west of SPM, which likely strongly affects CSW propagation, possibly inducing reflections, scattering into higher modes, generation of evanescent waves and mean currents^35^. These changes may result in a local mode 1 (in the cross-shore direction) ITW propagating anti-cyclonically around SPM. SPM may thus be nearly resonant at the O1 period, with an azimuthal mode 2 and cross-shore mode 1 ITW propagating around the archipelago, in a fashion similar to Rockall Bank (North Atlantic Ocean)^25^, or to Lastovo Island in the Adriatic Sea^4^. Figure 6f shows that K1 seems to be slightly farther away from azimuthal resonance, thus explaining the relative amplification of O1 with respect to K1. Interestingly enough, because these ITWs are predicted to be only weakly dispersive, an azimuthal mode 1 ITW would have a ~2 day period, which is indeed a weak frequency peak observed in the temperature and velocity spectra (see above and Figs 2c and 3a).

Nevertheless, these estimations are rather crude and, apart from the complicated structure of the forcing and of the archipelago, other dynamical mechanisms are likely to affect the propagation of these waves. An indication of this is for example the anomalous behaviour observed in P60 with respect to P30 (strong positive anomaly on 7 September 2011) or in M9E (strong negative anomaly on 14 September 2016). For example, interactions of a barotropic sub-inertial tide with the topography in a stratified environment may also give rise to “large amplitude unsteady lee waves”, as shown in the Kuril Straits^36^. In the cross-shore direction, some shoaling of the diurnal internal tide may also occur, as shown by the phase discrepancies between P30 and P60 (Fig. 3b). The drop in seabed temperature is induced by an upslope flow of cold water and seabed temperature rises when this cold water recedes. The speed at which the water cools and the time to recovery indicate the nature of this frontal intrusion and help determine whether it has the characteristics of a wave-like or a bore-like structure. Canonical cases feature a sharp drop in temperature (less than about 1 h) and a longer warming period that occurs over several hours and lasts until the water column returns to near homogeneity^37,38^. This type of structure reflects the breaking of the internal wave, and the cross-shore propagation of a strong leading wave followed by a gradually decreasing tail. A canonical case occurs when the Irribaren number (ratio of the bottom slope to the internal wave steepness) is low^39^. Conversely, a non-canonical case first shows a sharp, but time-limited, drop and continuous cooling for several hours followed by quick warming. From our observations, we cannot conclude as to which case is dominant. Both situations were observed (Fig. 2b), even during a single day (for example, on 3–4 September). We therefore assume that the prerequisites of internal wave propagation are subject to other factors, such as a change in stratification or nearshore circulation. For instance, the (Eulerian) residual current (Fig. 3b) was quite strong (>0.1 m/s) and of the order of magnitude of the O1 diurnal current. It is thus likely to flush out waters in each diurnal tidal cycle.

Semi-diurnal temperature fluctuations, although not dominant, are likely to result from internal wave propagation over a sloping bottom, as usually observed near strong bathymetric slopes. Nevertheless, the harmonic analysis of bottom temperature did not show clear evidence of propagation for the main semi-diurnal component M2 (see Supplementary Table S2). Semi-diurnal currents are shown to be of the same order of magnitude as diurnal tidal currents (at least in the Miquelon Bay). The fact that the semi-diurnal component of the seabed temperature variability is weak in comparison with diurnal variability may be due to the scattering of the semi-diurnal internal wave, which is able to propagate freely and away from the archipelago in this region.

We reported high-amplitude daily temperature fluctuations (up to 11.5 °C) observed near the bottom at 30–60 m depth, possibly the largest oscillations ever observed — at any frequency — on a stratified mid-latitude continental shelf. These observations were based on water column velocity and temperature profiles in Miquelon Bay and an array of bottom temperature sensors around the SPM archipelago. Simple physical interpretation and modelling support the observed oscillations. Our analysis points to the amplification of diurnal bottom-intensified tidal CTWs/ITWs, resonant around the archipelago. Temperature oscillations are thought to be the manifestation of a CTW forced by the diurnal tide. In the cross-shore direction, this CTW may also give rise to a non-linear internal wave (NLIW)^37,40,41^.

## Methods

The bathymetry presented in Fig. 1 was extracted from the General Bathymetric Chart of the Oceans (GEBCO) with a 30 arc-second interval grid (http://www.gebco.net/data_and_products/gridded_bathymetry_data/).

The data set used for this study is described in Table 1.

Temperature (T) and salinity (S) time series and CTD casts (Fig. 1) were measured using NKE STPS probes. They have a time constant (the time required to rise or fall of around 63% (1 − e^−1^) between an old and new values of temperature) of less than 1 s and error of measurement of 0.05 °C and 0.1 psu.

Salinity profiles in Fig. 1d were based on a T/S relationship. A CTD cast at Station M9 in September 2016 led to the following relationship: S = −0.07* T° + 32.38 (R^2^ = 0.80). This relationship was applied to vertically (every 2 m) interpolated temperature from the bottom to the surface.

Mooring M9E is an extension of Mastodon system^42^ which aimed at measuring the temperature evolution from surface to the bottom. Each probe measured temperature and pressure and was housed in a small plastic bags filled with Marcol 82 insulating oil to reduce time constant. Previous tests have shown that this time constant lies between 30 second and 1 minute allowing an acquisition time step of 2 minutes. Vertical temperature profiles result from linear interpolation every 2 meters from the bottom between two probes by taking into account the vertical location of each probe measured by the pressure sensor. The near surface sensor was clamped below a small buoy (20cl) and then remained at the surface. This buoy was attached to the mooring with à 15 m long thin rope to the sub-surface main buoy (11 l of buoyancy) 3 m below the surface which stretched vertically the whole mooring. On Fig. 1d, for the sake of clarity only 3 h profiles are shown. The strong cold event which occurred on 14 September 2016 (see Fig. 2e) has been taken into account for the time averaged profile (thick blue line on Fig. 1d). Calculations made without data from this event have shown negligible difference on the average.

Time series of bottom temperature (Stations 1–19, M7-M12, N1-N9) were measured using Mastodon moorings^42^. Data were collected with a time step of 1 min and decimated to 15 min (time constant was 9 min), error of measurements was about 0.1 °C.

Spectral analysis in Fig. 2 was performed with 6 degrees of freedom and Hamming windows with 50% overlap. Confidence intervals were calculated using a chi-square variable analysis. The analysed time series extended from 01 Aug to 15 Oct 2011.

Axis rotation (Fig. 3): The P30 mooring was located in a small bay and the curved isobaths did not allow straightforward determination of the cross- and along-shore directions. By assuming that the temperature variations resulted primarily from advection of bottom water in the cross-shore direction, we screened for the best correlation between temporal variation in temperature and the bottom current by rotating the axis system. When the time series of the time derivation of bottom temperature and bottom current (2 mab) were filtered between 0.5 h and 30 h, the best correlation was obtained with a rotation of 20° in the positive direction and reached 0.6. The current spectrum given in Fig. 3a was performed with 2 degrees of freedom and Hamming windows with 50% overlap (3 windows of 36 days). The frequency resolution of 0.024 cpd allowed spectral separation between O1 and K1 (Δω: 0.073 cpd).

Vertical temperature profiles (Fig. 3b) result from linear vertical interpolations of 1 mab, 5 mab, 16 mab and 24 mab time series with a vertical sampling of 2 m and a time step of 15 min.

Harmonic analysis (Figs 3e and 5, Supplementary Table S1–S2) was performed using the t_tide Matlab package^31^.

Normalisation of temperature and sea-level time series is the ratio of the detrended series to the standard deviation (Fig. 4d).

The EOF analysis (Fig. 6d) was performed using Matlab. Velocity vectors were used as complex values. The variance explained by the first mode of the EOF analysis for temperature and complex velocities (which means that the along-and cross-shore velocities are at the same scale) were 95% and 91% respectively.

ITWs Brink’s programs were downloaded from the site http://www.whoi.edu/fileserver.do?id=60003&pt=2&p=35366 (on 22/08/2017). The density profile (Fig. 6e) was based on the mean profile plotted on Fig. 1d for depths from the surface to 70 m; below these depths, climatological data extracted from the GDEM database^43^ were used. Sensitivity to variations in density profiles was also tested. To get an estimation of the sensitivity of Brink’s model to bathymetry (Fig. 6f), calculations were performed for four different bathymetric transects (north-west, north-east (Miquelon Bay), south-east and south-west of the archipelago) with topography derived from GEBCO.

## Electronic supplementary material

Supplementary information
