# Supplementary material for: Large diurnal bottom temperature oscillations around the Saint Pierre and Miquelon archipelago
Source: Sci Rep. 2018 Sep 17;8:13882. doi: 10.1038/s41598-018-31857-w (PMC6141533; doi:10.1038/s41598-018-31857-w)
Supplement: Supplementary file 1 — Supplementary information [file 41598_2018_31857_MOESM1_ESM.docx]

**Supplementary Information for**

**Large diurnal bottom temperature oscillations around the Saint Pierre and Miquelon archipelago**

P. Lazure*,^a^, B. Le Cann^a^, M. Bezaud^a^

^a^LOPS (Laboratoire d'Océanographie Physique et Spatiale), UMR 6523 CNRS-Ifremer-IRD-UBO, Brest, France

| **Station-**  **Depth**  **(mab)** | **O1** | | | | **K1** | | | | **M2** | | | |
| --- | --- | --- | --- | --- | --- | --- | --- | --- | --- | --- | --- | --- |
|  | U_maj_  (cm/s) | U_min_  (cm/s) | Inc.(°) | ɸ (°) | U_maj_  (cm/s) | U_min_  (cm/s) | Inc.(°) | ɸ (°) | U_maj_  (cm/s) | U_min_  (cm/s) | Inc.(°) | ɸ (°) |
| P30-2m | 9.2 | -0.8 | 143 | 138 | 3.0 | -1.6 | 154 | 218 | 3.5 | -2.3 | 121 | 352 |
| P30-10m | 10.2 | -1.1 | 114 | 142 | 4.3 | -0.7 | 139 | 231 | 7.4 | -1.2 | 125 | 24 |
| P30-18m | 13.1 | -1.6 | 99 | 142 | 4.7 | 0.8 | 121 | 210 | 10.7 | -1.4 | 121 | 16 |
| P30-24m | 14.5 | -1.5 | 98 | 136 | 4.1 | 0.8 | 82 | 169 | 11.9 | -2.4 | 118 | 11 |
| L3-2m | 11.4 | -4.0 | 96 | 221 | 6.8 | -3.3 | 95 | 271 | 3.1 | 1.9 | 10 | 235 |
| L3-10m | 20.5 | -7.5 | 82 | 218 | 12.7 | -5.4 | 90 | 261 | 6.1 | 0.6 | 127 | 6 |
| L3-18m | 24.5 | -9.4 | 68 | 220 | 14.2 | -6.5 | 78 | 264 | 6.9 | -0.4 | 117 | 360 |
| L3-24m | 24.5 | -9.7 | 67 | 219 | 13.4 | -6.1 | 79 | 261 | 6.6 | -0.2 | 114 | 360 |
| L4-2m | 9.8 | 0.3 | 9 | 276 | 6.4 | -1.4 | 32 | 291 | 10.2 | 1.5 | 164 | 351 |
| L4-9m | 16.4 | -0.6 | 177 | 112 | 10.1 | -2.7 | 10 | 289 | 14.9 | 2.9 | 171 | 348 |
| L4-16m | 19.9 | -5.7 | 163 | 123 | 11.7 | -5.1 | 176 | 112 | 14.5 | 3.6 | 170 | 349 |
| L4-23m | 21.4 | -9.3 | 151 | 135 | 10.4 | -9.4 | 157 | 132 | 12.5 | 3.4 | 161 | 347 |

*Table S1: Tidal ellipse components at the 3 ADCP moorings P30, L3 and L4 (see Fig. 1 for their location) for the 3 main tidal components. Umaj, Umin, Inc, ɸ denote the semi-major axis, semi-minor axis, inclination (from East ) and Greenwich phase respectively. Tidal ellipses are presented for 4 depths above the bottom (mab). Sign of semi-minor axis indicates polarity of ellipse (positive anticlockwise, negative clockwise). Harmonic analysis has been performed for the period August-September 2011 for P30 mooring, 2 July 2017 to 30 August 2015 for L3 mooring and 17 September 2015 to 17 October 2015 for L4 mooring.*

| **Station** | **O1** | | **K1** | | **M2** | | **S2** | | **Var(%)** | **SNR(O1)** |
| --- | --- | --- | --- | --- | --- | --- | --- | --- | --- | --- |
|  | A (°C) | ɸ (°) | A (°C) | ɸ (°) | A (°C) | ɸ (°) | A (°C) | ɸ (°) |  |  |
| 2 | 0.2 | 185. | 0.1 | 228. | 0.1 | 120. | 0.1 | 236. | 30. | 22. |
| 6 | 0.7 | 228. | 0.4 | 306. | 0.3 | 128. | 0.1 | 142. | 76. | 517. |
| 8 | 1.1 | 296. | 0.4 | 1. | 0.2 | 324. | 0.1 | 131. | 41. | 247. |
| P60 | 2.1 | 26. | 1.1 | 57. | 0.4 | 111. | 0.2 | 237. | 62. | 13. |
| M12 | 0.2 | 42. | 0.2 | 128. | 0.1 | 34. | 0.1 | 297. | 21. | 0. |
| M11 | 0.4 | 84. | 0.4 | 145. | 0.4 | 344. | 0.1 | 28. | 40. | 25. |
| 14 | 0.6 | 121. | 0.4 | 158. | 0.5 | 354. | 0.1 | 42. | 43. | 69. |
| M9 | 0.2 | 120. | 0.1 | 147. | 0.1 | 155. | 0.2 | 138. | 14. | 5. |
| M8 | 0.1 | 203. | 0.1 | 52. | 0.3 | 211. | 0.1 | 244. | 16. | 5. |
| 18 | 0.2 | 231. | 0.1 | 279. | 0.0 | 25. | 0.0 | 52. | 25. | 72. |
| M7 | 0.2 | 350. | 0.2 | 30. | 0.2 | 242. | 0.0 | 285. | 23. | 37. |
| N3 | 0.7 | 323. | 0.1 | 34. | 0.3 | 51. | 0.0 | 352. | 32. | 190. |
| N5 | 0.1 | 167. | 0.1 | 134. | 0.1 | 159. | 0.2 | 217. | 29. | 1. |
| N7 | 0.3 | 339. | 0.3 | 40. | 0.2 | 102. | 0.1 | 202. | 27. | 50. |
| N9 | 0.2 | 325. | 0.2 | 76. | 0.4 | 253. | 0.2 | 341. | 41. | 106. |
| 1 | 1.1 | 115. | 0.8 | 141. | 0.3 | 129. | 0.2 | 262. | 24. | 25. |
| 3 | 1.1 | 174. | 0.7 | 217. | 0.2 | 91 | 0.1 | 287 | 23. | 135. |
| 5 | 0.4 | 267. | 0.2 | 300. | 0.1 | 144. | 0.1 | 197. | 39. | 171. |
| L3 | 0.5 | 260. | 0.4 | 300. | 0.3 | 158. | 0.1 | 146. | 09. | 224. |
| 7 | 3.0 | 271. | 1.4 | 325. | 0.5 | 92. | 0.0 | 170. | 56. | 296. |
| L4 | 2.0 | 347. | 1.4 | 0. | 0.5 | 24. | 0.5 | 76. | 32. | 60. |
| P30 | 1.7 | 53. | 0.7 | 86. | 0.4 | 174. | 0.2 | 307. | 52. | 66. |
| 11 | 2.5 | 68. | 1.4 | 97. | 0.3 | 278. | 0.1 | 22. | 48. | 10. |
| 13 | 2.1 | 121. | 1.1 | 172. | 0.5 | 39. | 0.2 | 86. | 48. | 58. |
| 15 | 1.2 | 156. | 0.6 | 237. | 1.0 | 171. | 0.3 | 239. | 29. | 22. |
| 19 | 1.5 | 44. | 1.4 | 104. | 0.9 | 7. | 0.5 | 81. | 54. | 15. |
| N1 | 0.8 | 103. | 0.1 | 53. | 0.7 | 209. | 0.1 | 68. | 31. | 4. |
| N2 | 2.8 | 261. | 1.2 | 360. | 0.5 | 119. | 0.3 | 144. | 48. | 156. |
| N4 | 0.8 | 191 | 0.2 | 269. | 0.9 | 210. | 0.3 | 275. | 31. | 9. |
| N6 | 1.2 | 331. | 0.7 | 149. | 0.4 | 31. | 0.3 | 250. | 20. | 40. |
| N8 | 0.3 | 18. | 0.3 | 88. | 0.6 | 271. | 0.3 | 40. | 26. | 1. |

*Table S2: Tide harmonic constants (phase and amplitude) computed with t_tide for the observed bottom temperature during July and August (September for L4 station). Only the 4 main waves : O1, K1, M2, S2 are presented. Last columns presents the ratio of the variance predicted by the harmonic analysis to the variance of the original series and the SNR (Signal/Noise Ratio). The colors of the station names are in accordance with the figure 1 map and figure 4a (i.e.: green and red for 30 m and 60 m station depth respectively)*
